# Spectrum of respiratory viruses identified from SARS-CoV-2-negative human respiratory tract specimens in Watansoppeng, Indonesia

**DOI:** 10.1099/acmi.0.000840.v3

**Published:** 2024-10-11

**Authors:** Irfan Idris, Isra Wahid, Ungke Antonjaya, Edison Johar, Fiqry Hasan Kleib, Ida Yus Sriyani, Aghnianditya Kresno Dewantari, Oderna Daming, Mustakim Duharing, Fatmawati Sappe, Hajar Hasan, Frilasita Aisyah Yudhaputri, Din Syafruddin, Khin Saw Aye Myint

**Affiliations:** 1 Faculty of Medicine, Universitas Hasanuddin, Makassar, Indonesia; 2 Oxford University Clinical Research Unit Indonesia, Faculty of Medicine, Universitas Indonesia, Jakarta, Indonesia; 3 Eijkman Institute for Molecular Biology, Jakarta, Indonesia; 4 Exeins Health Initiative, Jakarta, Indonesia; 5 Soppeng District Health Office, Soppeng, Indonesia; 6 Soppeng District Health Laboratory, Soppeng, Indonesia

**Keywords:** COVID-19, HMPV, influenza, Indonesia, respiratory viruses, RSV

## Abstract

Respiratory infections account for millions of hospital admissions worldwide. The aetiology of respiratory infections can be attributed to a diverse range of pathogens including viruses, bacteria and fungi. SARS-CoV-2 (severe acute respiratory syndrome coronavirus 2)-negative specimens from Wattansoppeng city, South Sulawesi, were analysed to study the spectrum of respiratory viruses. Samples were screened for influenza virus, enterovirus, Paramyxoviridae, Nipah virus, Coronaviridae and Pneumoviridae. Of 210 specimens, 19 were positive for respiratory syncytial virus (RSV)-A, RSV-B, human parainfluenza virus type 1 (HPIV-1), HPIV-2, human rhinovirus (HRV)-A, HRV-B, HRV-C, human metapneumovirus (HMPV), influenza A virus (IAV) and coxsackievirus A6 (CV-A6). Influenza virus was of seasonal H3N2 subtype. The HMPVs were of genotypes B1 and A2a, while one RSV-A was of the ON-1 genotype. The viruses mostly affected children with unknown severity.

## Data Summary

RSV and HMPV sequences were compared to the NCBI blast nucleotide database (https://blast.ncbi.nlm.nih.gov/Blast.cgi) and deposited in NCBI GenBank (https://www.ncbi.nlm.nih.gov/genbank/) with accession numbers PP683454, PP683455 and PP683456. The authors confirm all supporting data, code and protocols have been provided within the article.

## Introduction

Respiratory infections, notably in the lower respiratory tract, are responsible for high morbidity and mortality globally in young children and the elderly, even before the coronavirus disease 2019 (COVID-19) pandemic [1]. The aetiology of respiratory infections can be attributed to a diverse range of pathogens, including viruses, bacteria and fungi. Non-SARS-COV-2 (severe acute respiratory syndrome coronavirus 2) respiratory viruses continued to circulate globally as causes of respiratory illnesses throughout the COVID-19 pandemic [2] although they were not well documented in Indonesia.

Viruses are one of the main causes of respiratory infections, with many pathogenic viruses emerging from wildlife [3]. Human activities including land exploitation, climate change, bush meat consumption and industrial farming create an intensive human–animal interface that increases the likelihood of spillover events, such as Hendra virus spillover from bats in Australia [4]. Bats, a natural host for many emerging viruses with pandemic potential, have received increased scrutiny due to their capacity to host a number of coronaviruses and paramyxoviruses [5]. The present study aims to investigate the viral aetiology of respiratory illness from SARS-CoV-2-negative patients in Watansoppeng, South Sulawesi.

## Methods

**Fig. 1.**
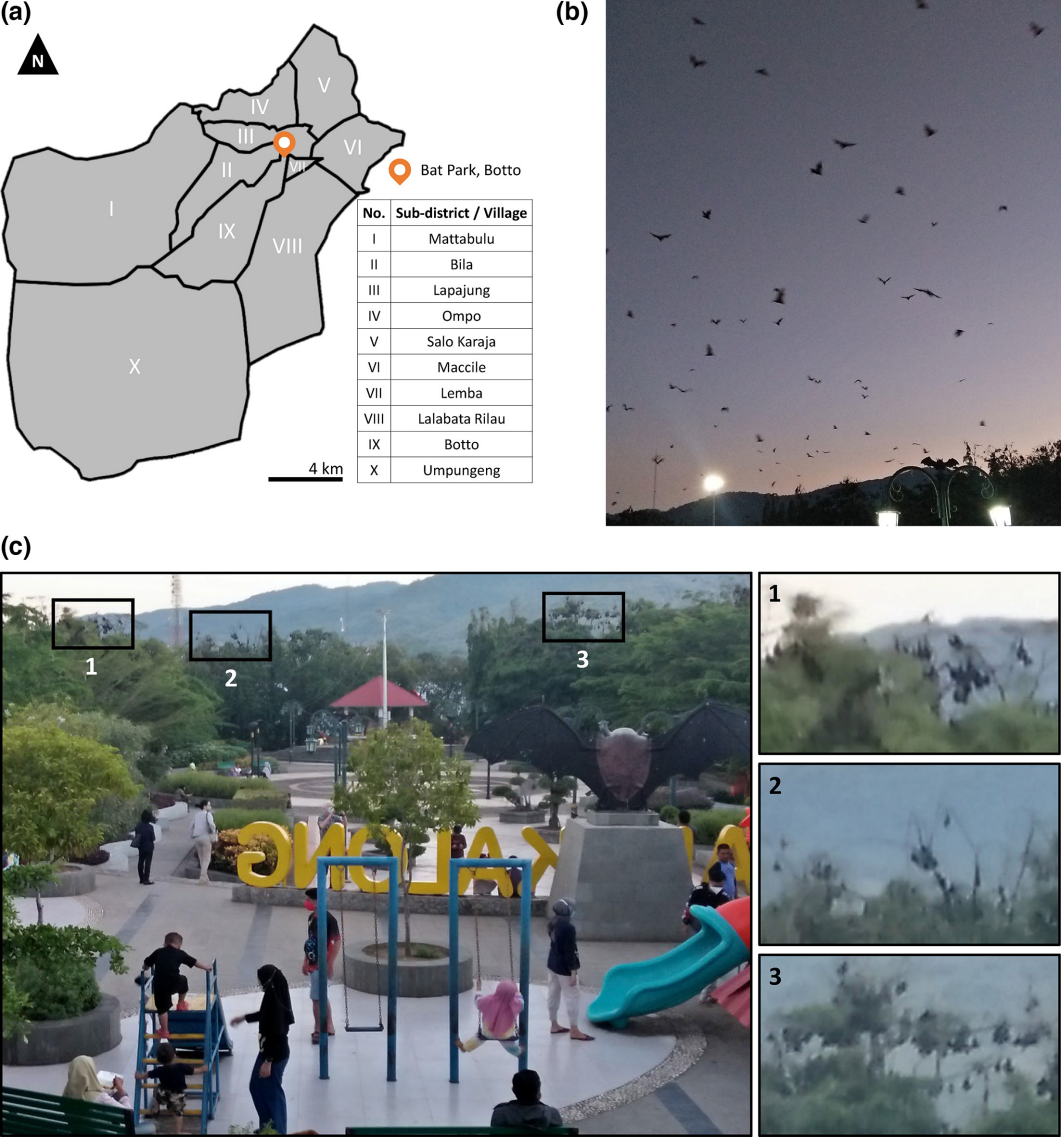
(**a**) Map of Lalabata district in Watansoppeng city and location of the bat park. (**b**) Bats flying at dusk. (**c**) Wattansoppeng bat park in the afternoon. Bat roosts in trees (image inserts). The photo in (**b**) is courtesy of Isra Wahid and (**c**) is courtesy of Ungke Antonjaya.

Watansoppeng city, Lalabata district, Soppeng regency, South Sulawesi, is located in a mountainous area with an altitude of 5–1500 m above sea level. Lalabata district has a population of 49  828 inhabitants in an area of 278 km^2^ (179 persons per km^2^) [6]. The city has a bat park in the city centre that serves as roosting area for *Acerodon celebensis* and *Pteropus alecto* fruit bats [7] (Fig. 1). Archived nasopharyngeal samples were collected from a previous SARS-CoV-2 surveillance initiative in Soppeng. Samples included from patients with suspected COVID-19 presenting with fever ≥38 °C and respiratory symptoms in puskesmas (primary healthcare centre) Cangadi, puskesmas Malaka, puskesmas Salotungo, puskesmas Tajuncu, rumah sakit unit daerah (district hospital) Latemmamala, and Soppeng unit pelaksana teknis dinas laboratorium kesehatan daerah (public health laboratory). Archived samples that were SARS-CoV-2 negative by a nucleic acid amplification test (NAAT), collected between March 2020 to July 2021, were shipped to Eijkman Institute for Molecular Biology, Jakarta.

The samples were further processed for NAAT screening with a battery of consensus respiratory virus panels at the genus level, such as for influenza virus and enterovirus, and at the family level such as for Paramyxoviridae, Coronaviridae and Pneumoviridae, and with Nipah virus-specific primers [8,9]. NAAT-positive samples were confirmed by Sanger sequencing. Samples that were positive for influenza A virus (IAV), respiratory syncytial virus (RSV) and human metapneumovirus (HMPV) were further characterized as previously described [10,12]. Both RSV and HMPV sequences were compared to the NCBI blast nucleotide database (https://blast.ncbi.nlm.nih.gov/Blast.cgi) and deposited in NCBI GenBank (https://www.ncbi.nlm.nih.gov/genbank/) with accession numbers PP683454, PP683455 and PP683456. This study was approved by the Eijkman Institute Research Ethics Commission (no. 127).

## Results

The spectrum of respiratory viruses was studied from 210 archived SARS-CoV-2-negative samples. The samples were collected from patients with a median age of 41 years old (range: 2 months to 96 years). Adults made up the majority of the patients (51.0%), followed by the elderly (26.7%), infants (13.3%) and children (9.0%). Gender was almost balanced with 53.3% females. The top three sub-districts where the patients resided were Lalabata Rilau (22.9%), Lapajung (16.7%) and Bila (15.7%) (Table 1).

**Table 1.** Demographics of the study population

Variable		*N* (% of total)
Age group(years old)	Median (range)	41 (0.2–96)
	Infant (<5)	28 (13.3%)
	Children (5–17)	19 (9.0%)
	Adults (18–59)	107 (51.0%)
	Elderly (>59)	56 (26.7%)
Gender	Male	98 (46.7%)
	Female	112 (53.3%)
Place of patient residence	Bila	33 (15.7%)
	Botto	27 (12.9%)
	Lalabata Rilau	48 (22.9%)
	Lapajung	35 (16.7%)
	Lemba	19 (9.0%)
	Maccile	16 (7.6%)
	Mattabulu	1 (0.5%)
	Ompo	15 (7.1%)
	Salo Karaja	13 (6.2%)
	Umpungeng	1 (0.5%)
	Others	2 (0.9%)

**Fig. 2.**
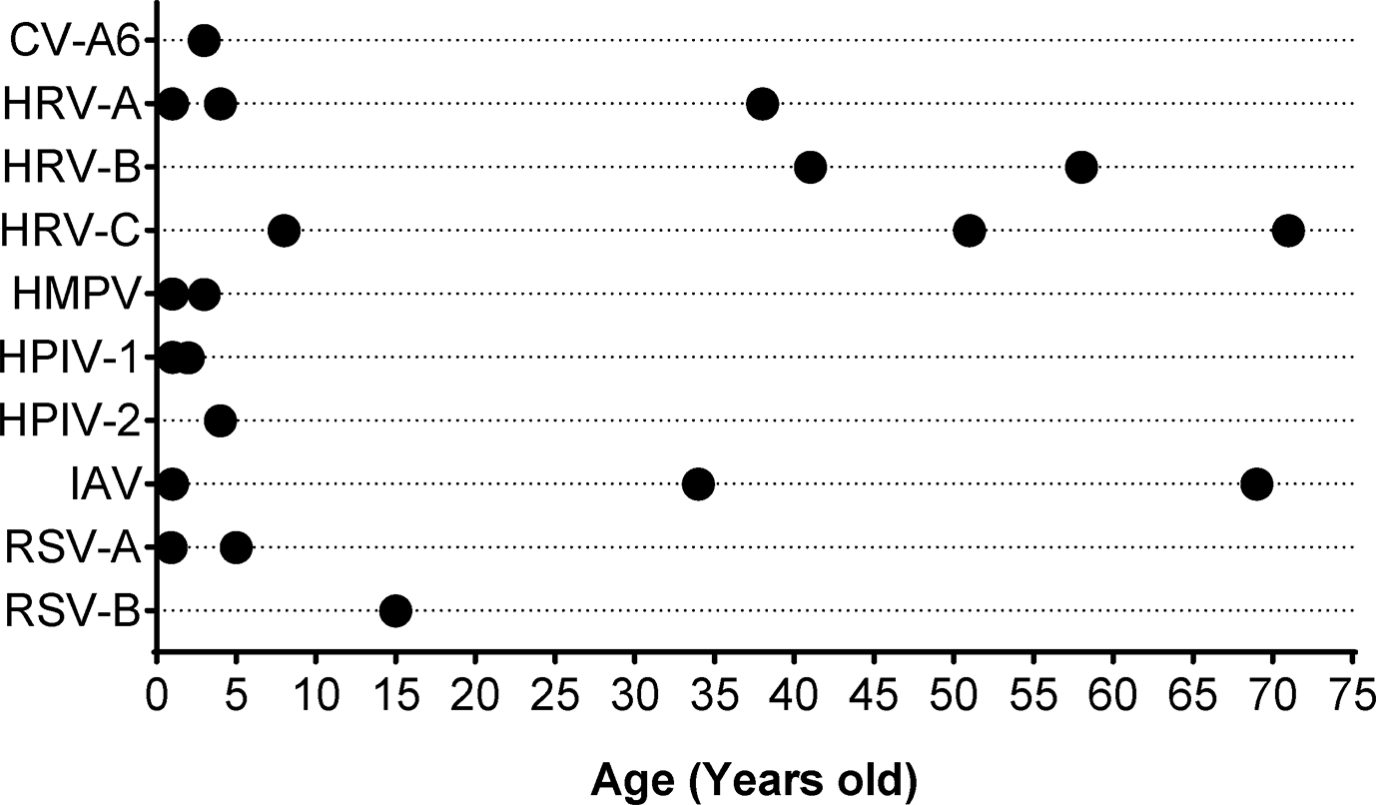
Age distribution of patients and virus species of positive cases.

Of 210 samples, 19 (9.1%) tested positive for ten virus species: RSV-A (*n*=2), RSV-B (*n*=1), human parainfluenza 1 virus (HPIV-1, *n*=2), HPIV-2 (*n*=1), human rhinovirus A (HRV-A, *n*=3), HRV-B (*n*=2), HRV-C (*n*=3), HMPV (*n*=2), IAV (*n*=3) and coxsackievirus A6 (CV-A6, *n*=1). One sample was positive for both HRV-A and HMPV. Moreover, 47.4% (9/19) of the positive samples were from infants (Fig. 2). It was observed that HRV and IAV infected several age groups, whereas the remaining virus species were confined to the paediatric population.

IAV, HMPV and RSV were further characterized and the influenza A viruses were found to be of seasonal H3N2 subtype. The HMPVs were of genotypes B1 and A2a. The genotype B1 isolate was genetically close to an isolate collected in 2018 from China with a similarity of 99.48% (accession no. MZ221202.1), whereas the genotype A2a isolate was most similar to a 2022 Australian isolate at 99.41% (accession no. OY757685.1). Only one of the RSV-A isolate’s G gene was successfully amplified, and it was of ON-1 genotype. The isolate was most similar to that collected in 2018 from China at 99.12% (accession no. OR666512.1). RSV-B characterization was unsuccessful.

## Discussion

The Indonesian ministry of health reported lower respiratory tract infections (LRIs) as the ninth leading cause of death in the country between 2007 and 2017, and the eighth leading disease burden in 2019; in Sulawesi, the burden of LRI was well above the national average [13]. In this study, we describe the spectrum of respiratory viruses causing COVID-19-like illness in an urban population in Wattansoppeng, South Sulawesi. HRV and IAV were identified as the most prevalent, whilst the spectrum of respiratory viruses was comparable to those found in Indonesia before the COVID-19 pandemic [14].

The distribution of respiratory viruses was similar to that of previous reports, mainly affecting children and the elderly [2,15]. HMPV, HPIV and enteroviruses including HRV and CV-A6 were more prevalent and detrimental in children, while RSV and IAV affected all ages [15,17]. Although HMPV, HPIV and HRV are mostly associated with common colds, CV-A6 is linked to hand, foot and mouth disease, and was detected in one child with unknown severity [18]. Interestingly, characterization of RSV-A revealed an emerging ON-1 genotype with global expansion [19]. The ON-1 genotype has been reported to increase severity of disease in children; mutations in the G protein have been linked to altered immunogenicity against the virus [19]. The seasonal influenza A/H3N2 is a common cause of respiratory infection of all ages. Influenza B, often reported in children, was not found in our study [8,14, 20].

Wild bats are a reservoir for many zoonotic viruses with pathogenic potential in humans and animals. A number of pathogenic bat-borne viruses have emerged in humans, such as SARS-CoV, Middle East respiratory syndrome coronavirus (MERS-CoV), avian influenza, Nipah virus and Hendra virus [21]. Evidence of Nipah virus, Hendra virus and bat coronaviruses has been reported in bats from different regions of Indonesia that could pose a potential risk of viral spillover [22,26]. Emergence of novel respiratory viruses with pandemic potential are closely linked to dense animal and human populations located in tropical areas [27]. Watansoppeng has a unique ecology where humans and bats cohabit, which has a potential for disease spillover [28]. Despite frequent exposure of city dwellers to wild bats, no pathogenic bat-borne respiratory viruses were detected in the study specimens.

There are some limitations to our study. (1) The real burden of non-COVID respiratory illnesses might be underestimated due to the relatively small sample size and the national COVID-19 surveillance strategy which might not include asymptomatic or mild infections. (2) The study lacked detailed clinical data to determine disease severity. (3) Spatial and temporal virus distribution could not be determined owing to the limited number of positive specimens. (4) With only a few viral panels utilized, the majority of specimens (90.9%) remained undiagnosed. Bacterial panels (*Haemophilus influenzae* and *Streptococcus pneumonia*) as well as metagenomics sequencing were not conducted in this study. (5) No bat specimens, which are a protected species in Watansoppeng, were collected for viral studies to determine if they were harbouring any of the viral species detected in the human respiratory samples.

Respiratory infections are a severe threat to global public health. Indonesia is a high-risk area for emerging pathogens due to its biodiversity; however, comprehensive studies of respiratory viruses including bat-borne viruses are limited. Our study describes the virus aetiology of non-COVID respiratory tract illnesses in Watansoppeng city, which has unique ecological conditions for potential viral spillover. Although we did not find any novel pathogens, the information obtained from this study provides insight into the prevalence of circulating respiratory viruses in the region. Additional studies are warranted in similar human–animal interfaces for organizing preparedness for possible future pandemics.

## Abbreviations

COVID-19: coronavirus disease 2019
CV-A6: coxsackievirus A6
HMPV: human metapneumovirus
HPIV: human parainfluenza virus
HRV: human rhinovirus
IAV: influenza A virus
LRI: lower respiratory infection
MERS-CoV: Middle East respiratory syndrome coronavirus
NAAT: nucleic acid amplification test
RSV: respiratory syncytial virus
SARS‑CoV‑2: severe acute respiratory syndrome coronavirus 2
